# Leukocyte-Platelet-Rich Fibrin in Bone Regeneration after Periapical Surgery: A 30-Month Follow-Up Clinical Report

**DOI:** 10.3390/reports7020032

**Published:** 2024-04-26

**Authors:** Hatim A. Qurban, Hatem Hazzaa Hamadallah, Mohammad A. Madkhaly, Muhannad M. Hakeem, Ahmed Yaseen Alqutaibi

**Affiliations:** 1 Restorative Dental Sciences Department (Endodontic), College of Dentistry, Taibah University, Al-Madinah Al-Munawwarah 42353, Saudi Arabia; hqurban@taibahu.edu.sa (H.A.Q.); mhakeem@taibahu.edu.sa (M.M.H.); 2 College of Dentistry and Hospital, Taibah University, Al-Madinah Al-Munawwarah 42353, Saudi Arabia; 3 Preventive Dental Science Department, College of Dentistry, Taibah University, Al-Madinah Al-Munawwarah 42353, Saudi Arabia; mmadkhaly@taibahu.edu.sa; 4 Substitutive Dental Sciences Department (Prosthodontics), College of Dentistry, Taibah University, Al Madinah 41311, Saudi Arabia; alqutaibitaibah@gmail.com; 5 Prosthodontics Department, College of Dentistry, Ibb University, Ibb 70270, Yemen

**Keywords:** endodontics, leukocyte-platelet-rich fibrin, L’PRF, bone regeneration, periapical surgery

## Abstract

Aim and background: Periapical lesions, which occur due to the infection and necrosis of dental pulp, are a significant dental pathology that poses risks to oral and systemic health. These lesions often require interventions such as root canal treatment or periapical surgery. Recent research has focused on the effectiveness of biocompatible materials, including mineral trioxide aggregate, bioceramics, and leukocyte-platelet-rich fibrin (L’PRF), in improving healing outcomes. This report presents the application of leukocyte-platelet-rich fibrin (L’PRF) derived from the patient’s autologous blood to enhance bone healing. Case description: A 61-year-old woman with well-controlled hypertension and good oral hygiene visited the dental clinic due to a painless swelling near her upper left central incisor. After examination, it was determined that she had a periapical granuloma. The patient underwent successful root canal retreatment and apical surgery, during which leukocyte-platelet-rich fibrin was applied. After 30 months, she experienced significant improvement with no symptoms and substantial bone regeneration. Conclusion: Clinical evidence and this case study indicate that leukocyte-platelet-rich fibrin (L’PRF) may enhance healing post periapical surgery. Further research, including more extensive and longer-term randomized trials, must confirm L’PRF’s effectiveness and refine treatment protocols. Clinical significance: L’PRF enhances bone healing post periapical surgery. Clinicians should consider integrating L’PRF in periapical surgeries, ensure diligent follow-up, and inform patients of its long-term advantages. Further randomized trials are needed to refine L’PRF clinical guidelines.

## 1. Introduction

Periapical lesions are pathological conditions at the apex of a tooth root due to pulpal infection and necrosis. These conditions can lead to significant bone destruction and persistent inflammation, making timely and effective treatment crucial [1]. Untreated lesions not only cause pain and the spread of infection but also pose a risk of systemic health issues due to ongoing oral infection [2]. The primary objectives of treatment are to eliminate the infection and promote the healing of the periapical tissue. Root canal treatment is the main method used to achieve these goals, which involves the removal of infected pulp, disinfection, shaping, and sealing the root canal to prevent further infection [1]. When nonsurgical methods fail or are not feasible, periapical surgery, such as apicoectomy, is considered. This surgery involves removing the lesion, resecting the apical root tip, and placing a retrograde filling to seal the root canal from the apex [3].

A study by Manila Chieruzzi et al. examined the mechanical effects of static loading on teeth that have undergone endodontic treatment and have been restored using fiber-reinforced composite (FRC) endodontic posts. The study assessed six FRC posts, each featuring different types of fiber materials and two types of cement (one with primer and one without). The structural efficiency of these posts was evaluated under oblique forces that mimic masticatory loads. The mechanical testing results showed that these dental systems’ performance varied depending on the type of post material and cement used. When used with cement without primer, the conical glass fiber posts demonstrated the highest load resistance. Other post configurations, particularly carbon fiber posts in cement without primer, showed a significant increase in load resistance of 229% compared to those with primer. Furthermore, the fracture resistances of most systems exceeded typical masticatory loads. Scanning electron microscopy revealed the good adhesive properties of the second cement at dentine–post interfaces, confirming that the overall strength of these dental systems is significantly affected by the quality of the bonding at these critical junctions [4].

The use of biocompatible materials such as mineral trioxide aggregate (MTA) [5] and bioceramics [6] significantly enhances the efficacy of periapical surgery. These materials promote healing and bone regeneration by providing excellent sealing capabilities, reducing inflammation risk, and supporting faster periapical tissue regeneration compared to traditional materials. Additionally, their integration with surrounding tissues elicits a positive biological response and reduces the chances of infection recurrence. However, despite their advantages, it is essential to consider challenges such as handling issues, cost, potential discoloration, and the need for further long-term studies to maximize periapical bone healing [5,6].

Leukocyte-platelet-rich fibrin (L’PRF) and platelet-rich fibrin (PRF) are both autologous platelet concentrates that aid in bone regeneration, but they differ in composition and biological impact [7]. L’PRF, an advanced form of PRF, contains a higher concentration of platelets and leukocytes within its fibrin matrix. This higher concentration potentially enhances regenerative outcomes by delivering critical growth factors and cytokines for immune responses, inflammation control, and wound healing. Furthermore, the denser fibrin structure of L’PRF, resulting from its unique centrifugation process, may contribute to a sustained release of growth factors, thereby further supporting bone healing and integration [7,8,9].

Although the use of PRF in periapical surgery and its role in bone healing are well-documented [5,10,11,12,13,14,15,16], research on the specific impact of L’PRF remains limited. This clinical report aims to examine the potential of L’PRF derived from the patients’ own blood to improve bone healing post surgery.

## 2. Case Presentation

### 2.1. Chief Complaints History and Clinical Findings

**Figure 1:**
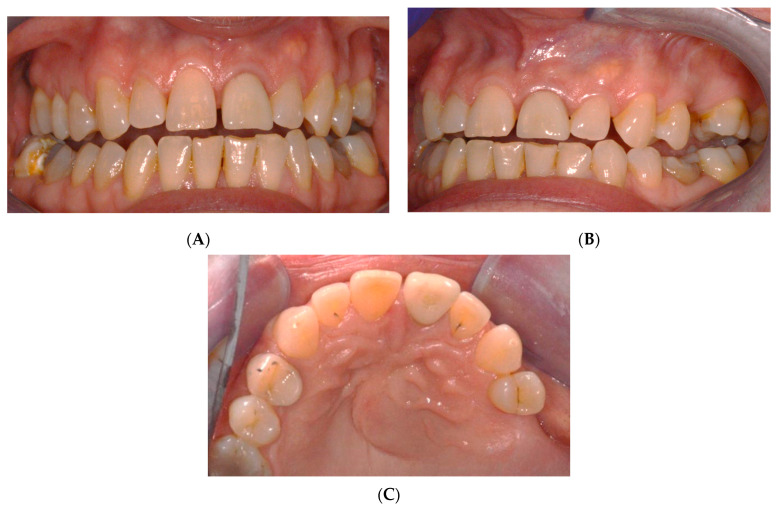
Pre-operative intraoral photos: (**A**) frontal, (**B**) lateral, and (**C**) occlusion views.

A 61-year-old female patient exhibited a non-painful swelling around the upper left central incisors that caused labial and palatal pressure (Figure 1). It was observed that the swelling had progressively increased in size during the lockdown period.

The patient presents a documented case of effectively controlled hypertension through the administration of Losartan potassium. Furthermore, the patient demonstrates excellent oral hygiene practices, as evidenced by their regular dental check-ups and the utilization of an electric toothbrush. The dental history of the patient indicates a consistent engagement with general dental practices, although they do not participate in interdental cleaning and do not use mouthwash. The patient uses an electric toothbrush (ETB) and fluoride toothpaste for their oral hygiene, which are positive preventative measures. The patient does not experience severe dental anxiety. In terms of oral health risks, the patient does not smoke. However, the dental examination revealed some concerns. The periodontal findings indicate some issues, with the Basic Periodontal Examination score in Region 4 suggesting pockets of 4–5 mm. The patient also showed tooth mobility in the upper left central incisors and lower right central incisor, both graded as Grade 1, indicating slight movement but not severe. There are no teeth tender to percussion or tender to prolonged palpation.

**Figure 2:**
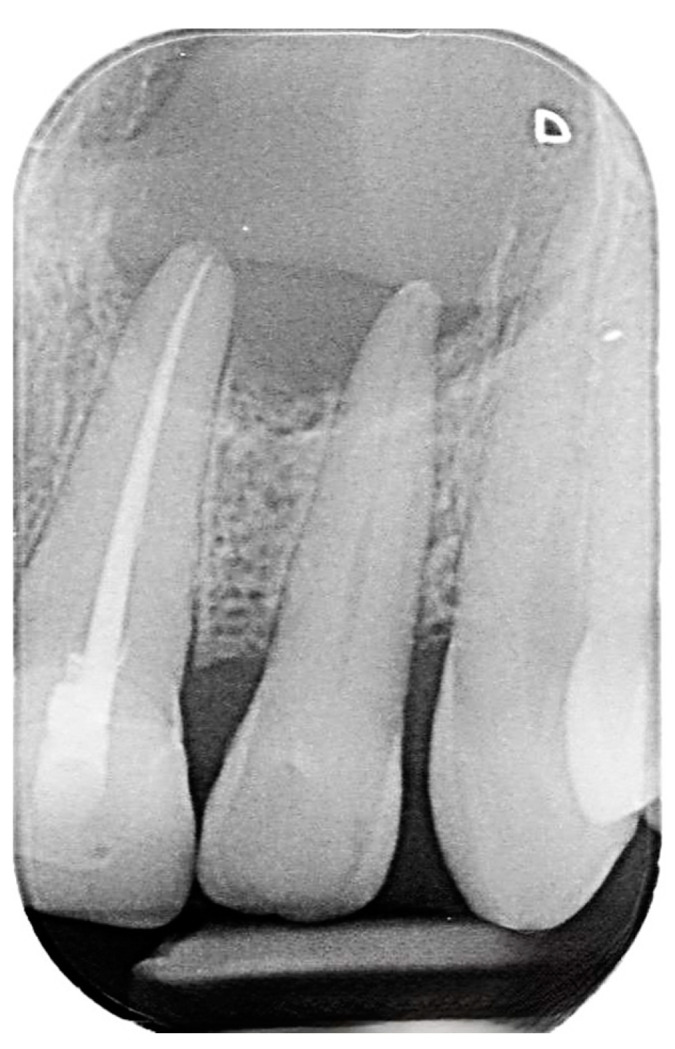
Pre-operative intraoral periapical radiograph of the upper left central and lateral incisors.

Despite having previously undergone root canal treatment and the placement of a crown on upper left central incisors in 2018, radiographic examinations have revealed suboptimal root canal filling and apical radiolucency in both left central and lateral incisors (Figure 2).

The extraoral examination revealed no abnormalities, while the intraoral examination was unremarkable, apart from the observed swelling. The patient became aware of the swelling during the lockdown period, and it progressively increased in size, although no pain was reported. Subsequently, a referral was made to an oral surgery department for a consultation. Following this, the patient was further referred to the restorative department to initiate a root canal retreatment before proceeding with apical surgery.

### 2.2. Delivery of Treatment

**Figure 3:**
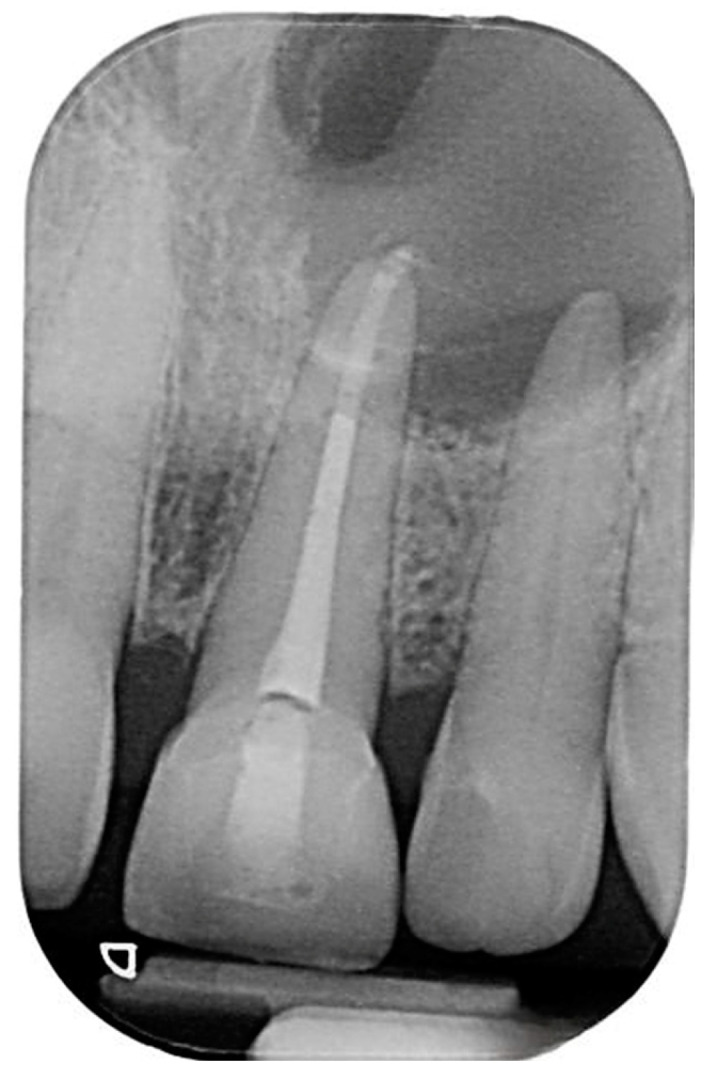
Postoperative intraoral periapical radiograph the upper left central and lateral incisors.

As previously mentioned, an exhaustive endodontic evaluation was conducted on the patient before proceeding with the root canal retreatment. The coronal restoration exhibits a clinically and radiographically acceptable margin. The decision was made to perform the retreatment procedure through the crown. Initially, an attempt was made to complete the root canal retreatment in a single visit. However, due to an inability to adequately dry the canal following physical and chemical debridement, it was necessary to dress the canal with calcium hydroxide. During the second visit, the canal was successfully dried. It was then obturated using an MTA plug and warm gutta-percha. Finally, the tooth was restored with a composite restoration (Figure 3).

After the completion of the root canal retreatment, the patient was referred back to oral surgery to undergo apical surgery. The patient provided her consent for the surgery, which involved the use of her own blood to obtain leukocyte-platelet-rich fibrin (L’PRF). A papilla base incision was made to alleviate the soft tissue, with a single vertical incision extending from the upper left canine to the upper right central incisor. Upon raising the flap, the cystic lesion was identified in the apical third of the upper left central incisors. In order to have the L’PRF prepared for placement in the surgical socket, the patient underwent cannulation to obtain four blood tubes prior to the complete enucleation of the cyst.

### 2.3. L’PRF Preparation

**Figure 4:**
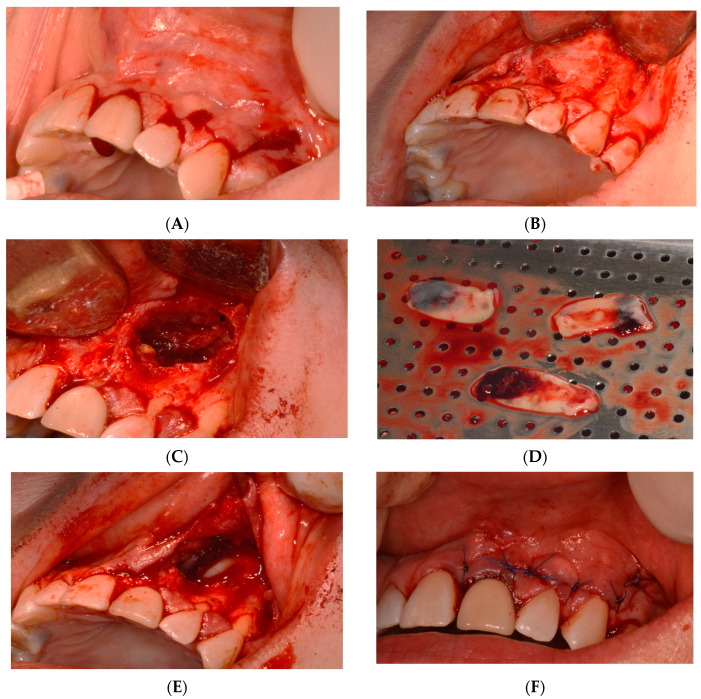
Intraoperative photographs: (**A**) incision, (**B**) flap reflection, (**C**) lesion excision, (**D**) LPR’f, (**E**) LPR’f placement, and (**F**) suture.

Membranes of leukocyte-platelet-rich fibrin (L’PRF) were generated by extracting a blood volume ranging from 10 to 40 milliliters, tailored to match the dimensions of the defect. The blood was collected in 9-milliliter tubes containing a clot activator as serum and then underwent centrifugation at a speed of 2700 revolutions per minute for 12 to 14 min. Following this centrifugation process, the resulting clot was transferred onto a designated tray for 10 min, during which the L’PRF membranes and plugs were formed. After the complete removal of the lesion, four L’PRF pieces were positioned within the surgical area. The first piece was placed on top of the socket, beneath the nasal cavity floor; the second was placed at the bottom of the socket; the third was centrally located within the socket; and the final piece was applied externally to cover the socket. Subsequently, the tissue flap was repositioned to its original location. Sustained pressure was applied for 5 min to facilitate initial stabilization, followed by securing the flap with 5-0 proline stitches (Figure 4).

**Figure 5:**
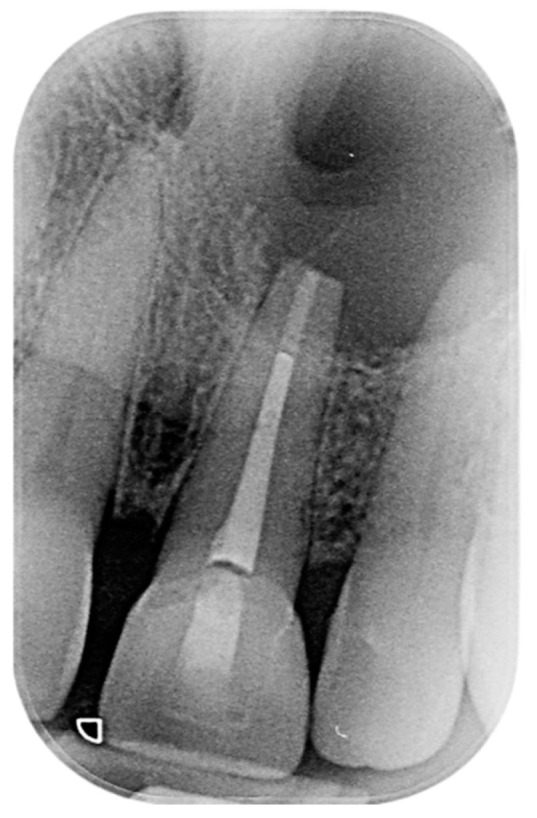
Post-surgery intraoral periapical radiograph of the upper left central and lateral incisors.

The cystic lesion ruptured during the enucleation procedure. Nevertheless, small fragments were collected and sent to the histopathology laboratory for examination (Figure 5). A post-surgery intraoral periapical radiograph was taken after the apical surgery and enucleation. The pathology report indicated that the CBCT scan revealed a radiolucency extending from the upper left central incisor to the upper left canine, with perforation into the nasal cavity, as well as palatal and buccal expansion. The upper left central incisor had previously undergone root canal treatment, and apical surgery was performed in conjunction with the enucleation of the lesion. A microscopic examination of the collected fragments revealed dense fibrous connective tissue and granulation tissue with neutrophils and vessels. No epithelial lining was observed. Additionally, areas of crushed minor salivary gland and benign cartilage were present, likely originating from the submucosa near the maxilla. These features were consistent with a diagnosis of periapical granuloma, with no signs of malignancy.

### 2.4. Healing of the Defect and Follow Up

**Figure 6:**
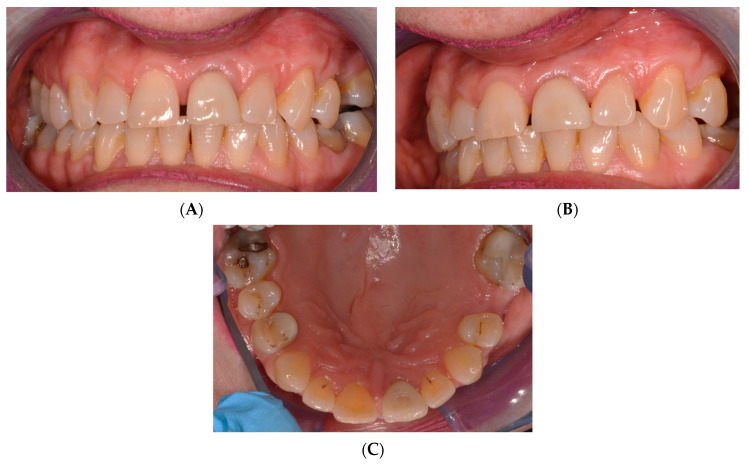
One-year-review, post-surgery intraoral photos: (**A**) frontal, (**B**) lateral, and (**C**) occlusion views.

The patient was evaluated after 12 months, 18 months, and then after 30 months. From a clinical standpoint, the patient did not exhibit any symptoms related to the tooth or the surrounding area. Following a clinical examination of upper right central and left lateral incisors, no indications of infection or inflammation were observed. The teeth did not elicit any tenderness upon periapical palpation utilizing a dental mirror handle or periodontal probe. Mobility was absent, and the probing depths around each tooth fell within the normal range, with no pockets exceeding 3 mm. The soft tissue around the teeth and surgical site was also assessed using a periodontal probe, revealing its healthy condition without any signs of redness, swelling, or tenderness (Figure 6).

**Figure 7:**
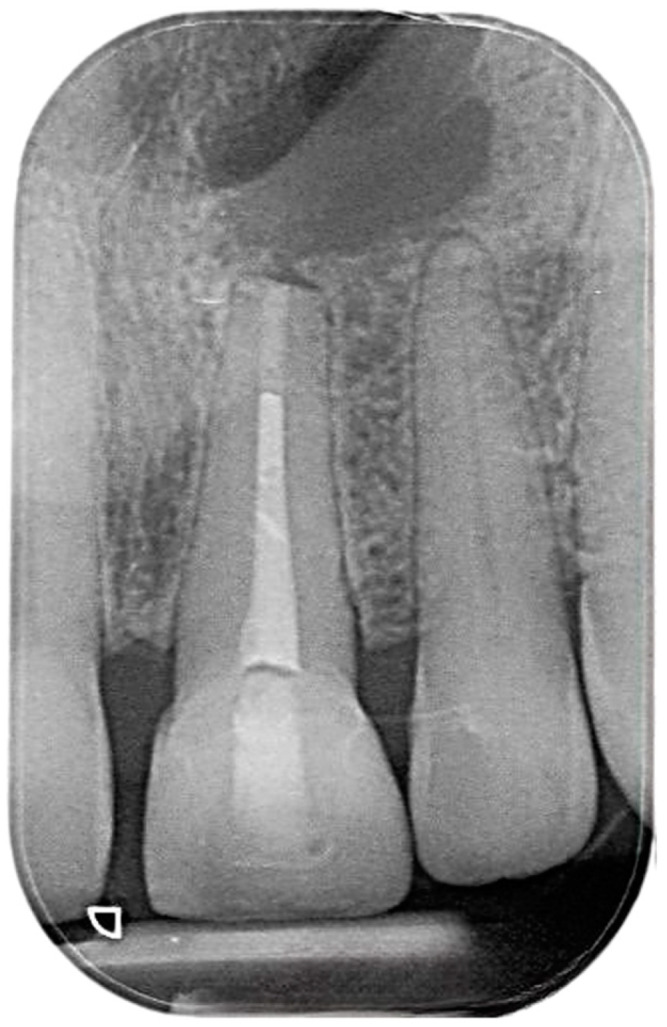
One-year-review, post-surgery intraoral periapical radiograph the upper left central and lateral incisors.

Vitality tests were conducted on the upper right central and left lateral incisors, utilizing both cold testing with ethyl chloride spray and electric pulp testing (EPT) with a pulp tester. To insulate it, a finger cot was placed over the patient’s finger for the EPT. Within 2 s, both teeth displayed a response, indicating their vitality. Conversely, periapical radiographs were obtained using the parallel technique and an X-ray positioning device to standardize the angle. Upon examination of the radiographs, a noticeable bone deposition was observed around the root apex of left central and lateral incisors. Furthermore, the previously observed lesion in the periapical area had significantly decreased in size (Figure 7).

**Figure 8:**
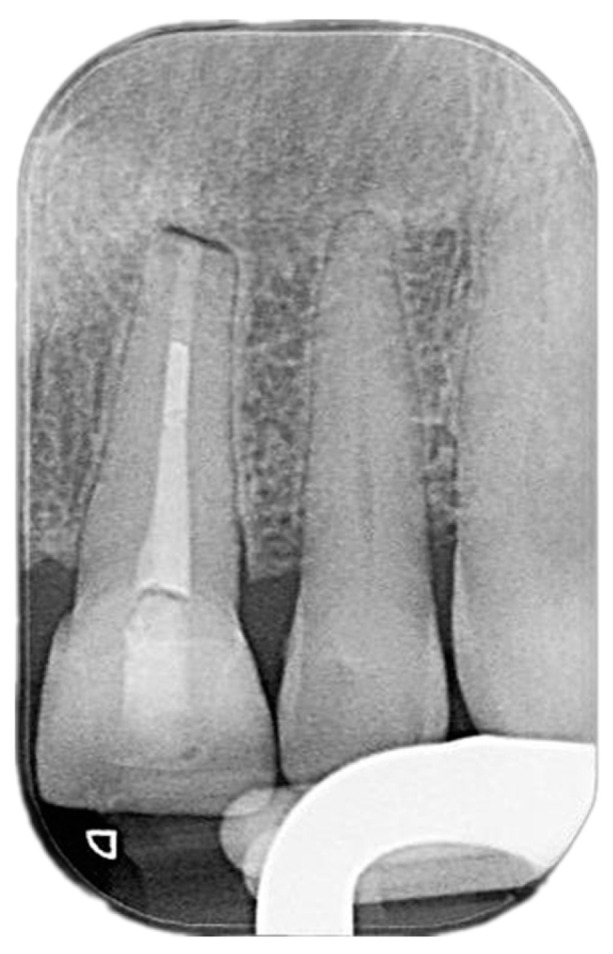
Two-and-a-half-year-review (30 months) post-surgery intraoral periapical radiograph of upper left central and lateral incisors.

The patient was reevaluated after 2.5 years (30 months). Clinically, no symptoms related to the tooth or the area were observed, suggesting an ongoing, smooth healing process. Upon clinical examination from right central to the left lateral incisors, there was no evidence of infection or inflammation. The radiograph demonstrated a further decrease in the size of the previous periapical lesion, accompanied by increased bone formation around the root apex of the teeth (Figure 8).

**Figure 9:**
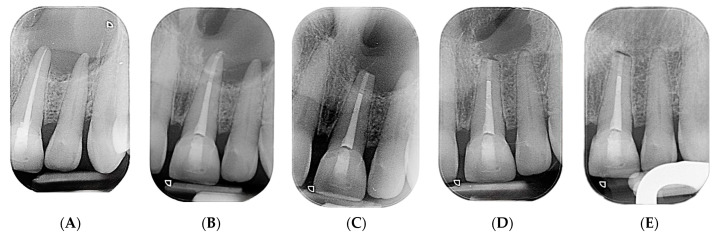
(**A**) Periapical radiograph taken before endodontic treatment, (**B**) periapical radiograph taken after nonsurgical root canal treatment, (**C**) immediate postoperative periapical radiograph following apical surgery, (**D**) one-year postoperative periapical radiograph, and (**E**) periapical radiograph from 30 months post operation.

Periapical radiographs were taken at 5 different times: pre endodontic treatment, post endodontic treatment, immediately after apical surgery, one year post surgery, and 30 months post surgery. The initial x-ray before treatment revealed a well-defined periapical radiolucency surrounding the apex of left central and lateral incisors. Despite undergoing nonsurgical root canal treatment, the lesion persisted. However, following surgery, there was a noticeable reduction in the size of the radiolucency. The bone began to occupy the previously affected area at the one-year mark. By the one-and-a-half-year mark, there was clear evidence of substantial new bone formation, accompanied by a notable reduction in the size of the previous radiolucency (lesion about 65% healed). At the 2.5-year follow-up, significant bone healing was evident in the radiograph (lesion about 95% healed). These radiographs illustrate the gradual healing progression of the periapical lesion through the deposition of bone after apical surgery. Moreover, they provide compelling evidence of the successful resolution of the pathology (Figure 9).

## 3. Discussion

Based on the clinical and radiographic findings, the priority for this case was to perform a root canal retreatment on the left central incisor before considering apical surgery. This approach is in line with the recommendation of Moiseiwitsch and Trope, who suggest starting with nonsurgical treatments due to their high long-term success rates [17,18]. They also mention that when a periapical lesion exceeds 20 mm, a combination of nonsurgical treatment and apical surgery may be necessary [17].

In this case, the left maxillary central incisor showed significant apical radiolucency, indicating unsatisfactory previous treatment. Therefore, a combined approach of nonsurgical root canal retreatment and apical surgery was chosen. The existing all-ceramic crown on the left central incisor was kept during the nonsurgical retreatment because it was intact, had satisfactory margins, and matched the appearance of the opposite tooth, as confirmed by clinical and radiographic evaluation. Recent studies support the effectiveness and high success rate of such procedures performed through intact crowns, with a success rate of 95.3% in treated cases. Failures were not due to endodontic reasons [19].

An MTA plug was used in the apical third of the canal to manage the wide apex, as this technique has been shown to have high success (94.6%) and survival rates (97.1%) [20]. This choice simplified the apical surgery procedure and reduced the time needed for retro-preparation.

The incision used for surgical access was a papilla base incision, which is recommended for anterior regions to minimize gingival recession and facilitate flap re-approximation and suturing [18,21]. After removing the cystic lesion, the surgical site was filled with L’PRF, which mainly consists of platelets and promotes healing by secreting growth factors [22,23,24]. The application of L’PRF, especially in large lesions, can improve healing outcomes and reduce postoperative discomfort [25].

A 5-0 proline suture was used for wound closure due to its monofilament nature, which reduces the risk of infection by preventing plaque accumulation, unlike braided or multi-strand sutures [26]. The sutures were removed after one week, although guidelines suggest their removal can occur 2 to 3 days after surgery [27,28,29].

Initially, the periapical lesion was diagnosed as either a radicular cyst or periapical granuloma, which are equally likely and commonly associated with the upper anterior teeth [30]. Subsequent laboratory analysis confirmed a periapical granuloma.

During the patient’s 30-month follow-up, there were no clinical symptoms, signs of infection or inflammation, abnormal probing depths, or tenderness upon palpation. Teeth right central incisor and left lateral incisor responded positively to vitality tests. Radiographic evidence indicated bone regeneration around left central and lateral incisors, which suggests ongoing healing, particularly significant given the initial size of the lesion.

Despite the widespread success of dental implants as a reliable solution for replacing missing teeth [31,32,33,34,35], there is a strong argument for keeping and restoring endodontically treated teeth instead of choosing extraction and subsequent implant placement [36]. When properly managed, root-canal-treated teeth can maintain both their function and appearance, preserving their natural tooth structure and alignment within the mouth. This approach avoids the surgical risks and potential complications associated with implants, such as peri-implantitis or implant failure. Additionally, retaining natural teeth helps to preserve the bone and surrounding tissue, which can deteriorate after extraction. There have also been significant advancements in endodontic treatments, improving patient success rates and outcomes. By treating and keeping these teeth, dental professionals can take a more conservative approach that respects the body’s natural biology, potentially providing patients with a simpler, less invasive, and cost-effective alternative to implants [36,37].

## 4. Clinical Applications

Based on the outcome of this clinical report, the following clinical methods are suggested for dental practitioners:

1. Utilize Leukocyte-Platelet-Rich Fibrin (L’PRF) in Periapical Surgery:
  ∙ When periapical surgery is indicated, consider using L’PRF as a biocompatible material to promote healing and bone regeneration.
  ∙ Prepare L’PRF from the patient’s own blood to minimize the risk of rejection and infection.
2. Manage Periapical Cysts with Combined Endodontic and Surgical Approaches:
  ∙ Treat persistent periapical lesions with a combination of a root canal treatment and periapical surgery for effective management.
  ∙ Employ modern materials like MTA for obturation and use L’PRF during apical surgery to enhance healing.
3. Monitor Healing Progress with Clinical and Radiographic Follow-ups:
  ∙ Schedule regular follow-up appointments post surgery to monitor the healing process through clinical examination and radiographs.
  ∙ Consider annual follow-ups for up to 3 to 4 years to ensure complete healing and adhere to post-apical-surgery guidelines.
4. Emphasize the Patient’s Quality of Life Post Surgery:
  ∙ Educate the patient about the potential for improved quality of life following the use of L’PRF in periapical surgeries.
  ∙ Inform patients that while their immediate postoperative quality of life may not significantly differ with L’PRF, its long-term benefits for bone healing are promising.

By integrating these clinical applications into practice, dental practitioners can potentially improve periapical surgery outcomes, enhance bone regeneration, and optimize patient care. It is also important to stay abreast of ongoing research and evolving techniques in the field to provide evidence-based treatments.

## 5. Limitations

This report does not adequately address whether L’PRF offers superior benefits compared to conventional PRF, which is crucial for establishing the clinical relevance of L-PRF. This issue is further complicated by the study’s design, which, being a single case report, lacks the statistical power to generalize its findings to a larger population or make definitive comparisons. Given the prevalence of periapical lesions in dental pathology, stronger evidence is needed, typically derived from randomized controlled trials or larger cohort studies, to determine the true effectiveness of L’PRF. Therefore, while the case report shows promising initial results, these limitations highlight the necessity of more comprehensive research designs and thorough methodological documentation to substantiate the advantages of L’PRF over PRF.

## 6. Conclusions

The evidence from the clinical reports and literature reviewed in this study suggests that leukocyte-platelet-rich fibrin (L’PRF) may potentially affect the healing process after periapical surgery. Moreover, a case study of a 61-year-old patient’s left central incisor demonstrated significant bone regeneration and positive clinical outcomes 16 months after using L’PRF, thus emphasizing its potential effectiveness in clinical settings. Based on these findings, it is crucial to carry out additional research efforts to improve treatment protocols and gain a thorough understanding of the role of L’PRF in bone healing after periapical surgery. Therefore, it is necessary to conduct additional randomized controlled trials with larger sample sizes and more extended follow-up periods to establish conclusive results regarding the effectiveness of L’PRF and its position in periapical surgery.

## Data Availability

The data are available upon request from the authors.
